# Predictive modeling of lower extreme deep vein thrombosis following radical gastrectomy for gastric cancer: based on multiple machine learning methods

**DOI:** 10.1038/s41598-024-66754-y

**Published:** 2024-07-08

**Authors:** Haiyan Zhou, Yongyan Jin, Guofeng Chen, Xiaoli Jin, Jian Chen, Jun Wang

**Affiliations:** 1 Nursing Department, The Second Affiliated Hospital, Zhejiang University School of Medicine, Hangzhou, 310000 Zhejiang China; 2 Department of Gastroenterology Surgery, The Second Affiliated Hospital, Zhejiang University School of Medicine, Hangzhou, 310000 Zhejiang China

**Keywords:** Gastric cancer, Gastrectomy, Deep vein thrombosis, Risk model, Machine learning, Gastric cancer, Computational biology and bioinformatics

## Abstract

Postoperative venous thromboembolic events (VTEs), such as lower extremity deep vein thrombosis (DVT), are major risk factors for gastric cancer (GC) patients following radical gastrectomy. Accurately predicting and managing these risks is crucial for optimal patient care. This retrospective case‒control study involved 693 GC patients from our hospital who underwent radical gastrectomy. We collected plentiful and comprehensive clinical indicators including a total of 49 baseline, preoperative, surgical and pathological clinical data. Using univariate logistic regression, we identified potential risk factors, followed by feature selection through the Boruta algorithm. We then constructed the final predictive model using multivariate logistic regression and evaluated it using receiver operating characteristic (ROC) curve analysis, calibration plots, decision curve analysis, and other methods. Additionally, we applied various machine learning techniques, including decision trees and random forests, to assess our model’s predictive strength. This retrospective case‒control study involved 693 GC patients from our hospital who underwent radical gastrectomy. We collected plentiful and comprehensive clinical indicators including a total of 49 baseline, preoperative, surgical and pathological clinical data. Using univariate logistic regression, we identified potential risk factors, followed by feature selection through the Boruta algorithm. We then constructed the final predictive model using multivariate logistic regression and evaluated it using receiver operating characteristic (ROC) curve analysis, calibration plots, decision curve analysis, and other methods. Additionally, we applied various machine learning techniques, including decision trees and random forests, to assess our model’s predictive strength. Univariate logistic analysis revealed 14 risk factors associated with postoperative lower limb DVT. Based on the Boruta algorithm, six significant clinical factors were selected, namely, age, D-dimer (D-D) level, low-density lipoprotein, CA125, and calcium and chloride ion levels. A nomogram was developed using the outcomes from the multivariate logistic regression analysis. The predictive model showed high accuracy, with an area under the curve of 0.936 in the training set and 0.875 in the validation set. Various machine learning algorithms confirmed its strong predictive capacity. MR analysis revealed meaningful causal relationships between key clinical factors and DVT risk. Based on various machine learning methods, we developed an effective predictive diagnostic model for postoperative lower extremity DVT in GC patients. This model demonstrated excellent predictive value in both the training and validation sets. This novel model is a valuable tool for clinicians to use in identifying and managing thrombotic risks in this patient population.

## Introduction

Gastric cancer (GC) remains one of the most common and deadly cancers worldwide, particularly among older males. According to GLOBOCAN 2018 data, GC is the fifth most common neoplasm and the third most deadly cancer, with an estimated 783,000 deaths in 2018^1^. Radical gastrectomy remains the main treatment for GC^2^. Patients who undergo GC surgery are at heightened risk for venous thromboembolic events (VTEs), including deep vein thrombosis (DVT) and pulmonary embolism (PE). The presence of VTE in cancer patients is a common complication and is considered a major health concern^3,4^. The VTE is also an independent predictor of death among patients with GC, with an overall mortality rate of 44.4% at 6 months postoccurrence^5^. Thus, there is a critical need for effective prediction and management strategies for VTE in postoperative GC patients.

Several specific mechanisms by which cancer influences VTE have been recognized. These tumors can be broadly categorized into two types: first, where the tumor alters systemic host factors such as platelets and leukocytes; and second, where the tumor secretes procoagulant substances into the bloodstream. These substances directly trigger the coagulation cascade or activate platelets^6^. The pathophysiology linking thrombosis and cancer is intricate. Malignancies are associated with a baseline hypercoagulable state, which contributes to the development of VTE^7^. Risk factors may include the type and stage of cancer, treatment modalities, and individual patient characteristics^8^. Understanding the risk factors for DVT and initiating appropriate prophylaxis are pivotal for reducing the risk of VTE^9^. Here, lower extremity DVT is the most common DVT among many DVT cases, especially in GC patients. The establishment of effective predictive models for postoperative lower extremity DVT in GC patients remains a challenge.

Predictive models play a crucial role in identifying patients at high risk of developing postoperative DVT. However, to date, very few predictive models for VTE specifically tailored to GC patients exist. One study reported strong performance and significant practical applicability in clinical settings, effectively pinpointing GC patients who are at an elevated risk for VTE^10^. One meta-analysis suggested that the risk of VTE following GC surgery is not notably high. Additionally, the risk of developing VTE is similar for patients who underwent either open or laparoscopic surgery for GC^11^. However, there are still no studies on the development of a predictive model for lower limb DVT after radical GC surgery.

The aim of our study was to develop and validate a predictive diagnostic model for lower extremity DVT formation in patients after radical surgery for GC. We sought to harness clinical and pathological data, potentially enhanced by machine learning algorithms, to predict the likelihood of DVT development. In this study, therefore, we aimed to fill an important gap in the current understanding and management of postoperative risks in GC patients.

## Materials and methods

### Data sources and study population

This was a retrospective case‒control study. Patients who underwent radical gastrectomy for GC between May 2020 and February 2023 at the Second Affiliated Hospital of Zhejiang University School of Medicine were selected as the study subjects. The inclusion criteria were as follows: (1) ≥ 18 years of age; (2) had a preoperative diagnosis of GC confirmed by gastroscopy and histological examination with subsequent radical gastrectomy and lymphadenectomy; (3) no evidence of distant metastasis in the liver, abdominal cavity, etc., as determined by preoperative imaging studies; (4) no history of DVT; (5) no severe disease affecting coagulation function and no use of medication influencing coagulation function; and (6) complete clinical data. The exclusion criteria for patients were as follows: (1) had a history of venous thrombosis; (2) had a history of coagulation dysfunction; (3) had distant metastasis preoperatively or intraoperatively; (4) had macroscopic or microscopic residual cancer cells at the surgical margins (R1/R2 resection); (5) had incomplete pathological diagnosis and clinical data; and (6) had a history of medication affecting coagulation function, such as aspirin, warfarin, or low-molecular-weight heparin. This study complied with the requirements of the Declaration of Helsinki, and all patients signed informed consent forms. The study protocol was reviewed and approved by the Human Research Ethics Committee of the Second Affiliated Hospital, Zhejiang University School of Medicine (Hangzhou, China).

### Diagnostic criteria for lower extremity DVT

The primary clinical symptoms of lower limb DVT following radical gastrectomy for GC include pain, swelling, and increased tension in the soft tissues of the affected limb. Our study adheres to the lower limb DVT diagnostic guidelines outlined in the “Venous thromboembolism in cancer patients” published by the ESMO Clinical Practice Guideline^12^. This guideline details the procedures for diagnosing VTE, encompassing both DVT and PE. In accordance with this guideline, we employed lower extremity venous Doppler ultrasound and plasma D-dimer level measurements. This study confirmed the absence of false positive results from the techniques used. Patients were categorized into two groups: those who developed lower limb DVT (the DVT group) and those who did not (the non-DVT group).

### Observation indicators

The collected baseline data included age, sex, body mass index (BMI), history of alcohol consumption, smoking history, history of allergies, preoperative comorbidities such as diabetes, hypertension or other underlying diseases, previous surgical history, and history of preoperative neoadjuvant chemotherapy. Preoperative clinical data included blood type; tumor markers such as carcinoembryonic antigen (CEA), alpha-fetoprotein (AFP), CA199, and CA125; total cholesterol (CHOL); triglycerides (TG); low-density lipoprotein (LDL); and high-density lipoprotein (HDL). Postoperative clinical data included white blood cell (WBC) counts, neutrophil counts (NE), hemoglobin (HGB), platelet (PLT), albumin (ALB), total bilirubin (TB), alanine aminotransferase (ALT), aspartate aminotransferase (AST), D-dimer (D-D), activated partial thromboplastin time (APTT), the international normalized ratio (INR), prothrombin time (PT), fibrinogen (Fib), potassium (K^+^), sodium (Na^+^), and chloride (Cl^−^) ions and calcium (Ca^2+^) ions. Surgical and pathological data included operation duration, blood transfusion volume, open/laparoscopic radical gastrectomy for GC, type of surgery (distal/proximal/total gastrectomy), tumor location (cardia/body/antrum), tumor size, histological grade (G1/G2/G3), lymphovascular invasion, perineural invasion, number of positive lymph node metastases, tumor infiltration depth (pT), and lymph node metastasis (pN). Therefore, a total of 49 clinical observation indicators were included in our study.

### Model development

All GC patients were randomly divided into a training set and a validation set at a 7:3 ratio, which is the most suitable sample size for this study. We first employed univariate logistic regression analysis to identify potential risk factors based on a *P* value < 0.05. Next, the Boruta algorithm was used to further filter the above variables. The Boruta algorithm is a feature selection method that is rooted in the theory of random forests; its primary objective is to identify genuinely important features from a given feature set while filtering out those that have no significant impact. By assessing the importance of features via a random forest and comparing them to randomized “shadow” features, the Boruta algorithm can be used to effectively address feature selection challenges. Finally, variables displaying statistical significance in the initial univariate analysis and Boruta algorithm were subsequently included in a multivariate logistic regression analysis, which was employed to identify the factors independently associated with lower extremity DVT and construct a predictive model.

### Model evaluation

Here, we employed various methods to assess the performance of the aforementioned predictive model, including discrimination, calibration, clinical practicability, multiple model comparisons, and multiple machine learning methods. To assess the discrimination and accuracy of the prediction model, the area under the receiver operating characteristic (ROC) curve (AUC) and calibration curve plot were generated. Additionally, decision curve analysis (DCA) was employed to evaluate the net benefits of the nomogram model at different threshold probabilities. Clinical impact curve (CIC) analysis was performed to evaluate the clinical applicability of the risk prediction nomogram. In addition, the net reclassification index (NRI), integrated discrimination improvement (IDI) and risk stratification were used to compare the nomogram with the traditional model system. Next, the predictive values of the nomogram model were compared with those of other models using multiple model ROC analysis. Finally, we applied various machine learning methods, including decision trees, random forests, support vector machines (SVMs), extreme gradient boosting (XGBoost), and light gradient boosting machines (LightGBMs), to determine the power of our predictive model.

### Data statistics and analysis

All the statistical analyses were conducted using SPSS software (version 27.0) and R software (version 4.2.2). For quantitative data, normally distributed measurements are expressed as the mean ± standard deviation (SD) and were analyzed using independent sample t tests. Normally distributed data are presented as medians and interquartile ranges, and nonparametric tests (Mann‒Whitney U test) were used for comparisons between groups. Categorical data are presented as numbers (%) and were analyzed using the chi-square (χ2) test. Univariate logistic regression analysis was used to identify risk factors for postoperative lower limb DVT in patients who underwent radical gastrectomy. Moreover, the Boruta algorithm was used for dimensionality reduction and variable selection. The variables selected through the above process were subsequently analyzed using multivariate logistic regression. A nomogram was developed to visualize the prediction model using the *rms* package. A P value exceeding 0.05 for the Hosmer–Lemeshow test indicated that the model’s fitness was sufficient. Multiple machine learning methods were performed in R language, including logistic regression (*glm* package), decision trees (*rpart* package), random forests (*randomForest* package), SVM (*e1071* package), XGBoost (*xgboost* package), and LightGBM (*lightgbm* package). In all analyses, a *P* value less than 0.05 was considered to indicate statistical significance.

## Results

### Baseline characteristics of lower extremity DVT after radical gastrectomy

**Figure 1:**
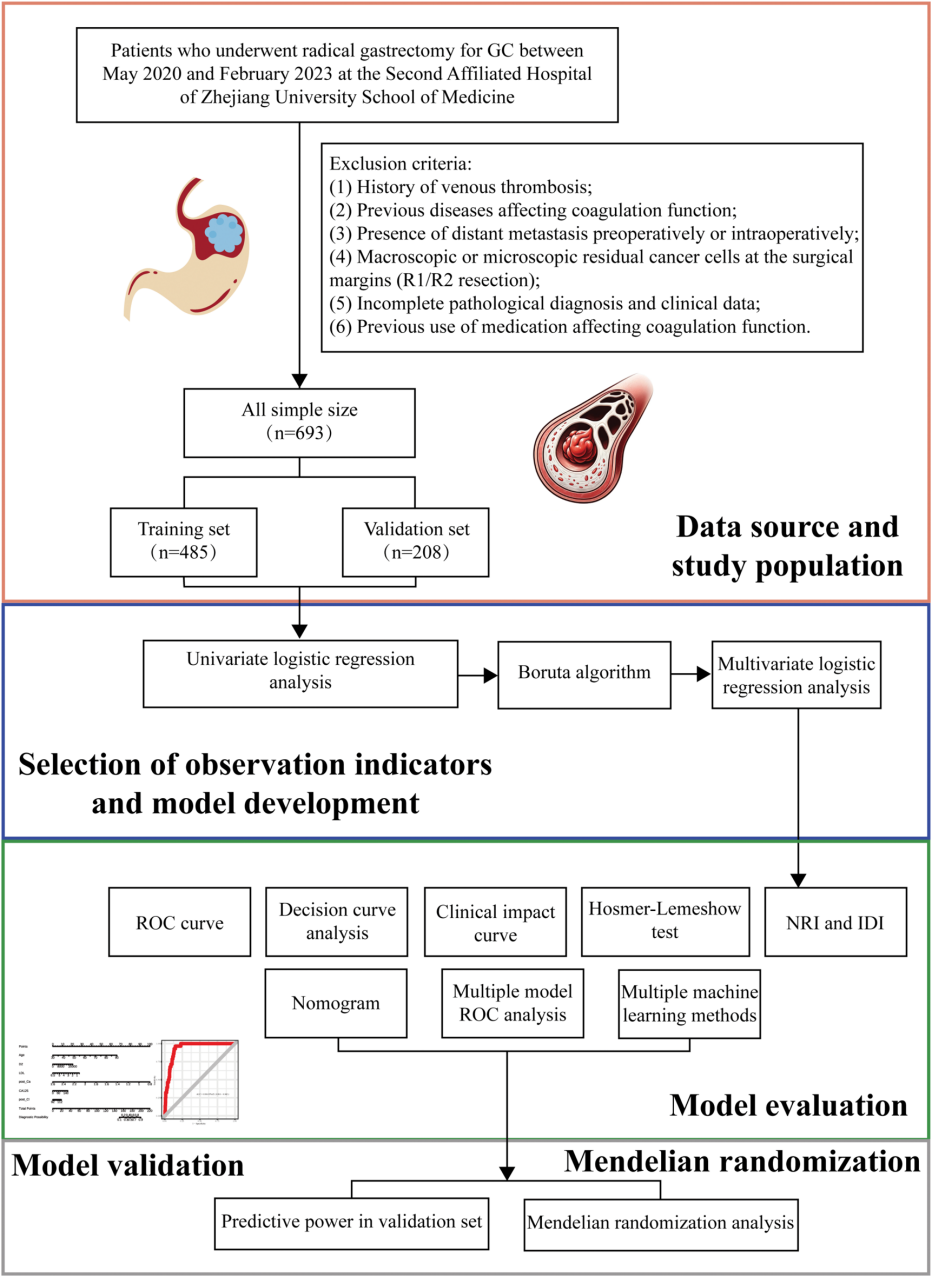
The flowchart of patient selection and study design.

In this study, a total of 693 patients who underwent radical gastrectomy for GC were included; 38 developed lower limb DVT, while 655 did not. The flowchart of patient selection and study design is shown in Fig. 1. The GC patients were randomly divided into a training set and a validation set at a ratio of 7:3, with 485 patients in the training set and 208 patients in the validation set. A total of 49 variables were included in this study, including age; sex; BMI; alcohol history; smoking history; allergy history; diabetes status; hypertension; other underlying medical history; previous surgical history; preoperative neoadjuvant chemotherapy history; blood type; CEA; AFP; CA199; CA125; CHOL; TG; LDL; HDL; WBC; NE; HGB; PLT; ALB; TB; ALT; AST; D-D; APTT; INR; PT; Fib; postoperative electrolyte levels; K^+^; Na^+^; Cl^+^; Ca^2+^; operation duration; blood transfusion volume; operation type (open/laparoscopic radical gastrectomy); surgical type (distal/proximal/total gastrectomy); tumor location; tumor size; histological grade (G1/G2/G3); lymphovascular invasion; perineural invasion; and number of positive lymph node metastases; and pT and pN. There were no statistically significant differences in the clinical baseline data between the training and validation groups (*P* value > 0.05; Supplementary Table 1).

### Establishment of a predictive model and nomogram for lower extremity DVT after radical gastrectomy

Next, we identified risk factors for lower limb DVT following radical gastrectomy. The training dataset (n = 485) included 28 patients with lower limb DVT and 457 patients without DVT following radical gastrectomy. Univariate logistic analysis revealed 14 risk factors associated with postoperative lower limb DVT, including age; blood transfusion volume; open/laparoscopic radical gastrectomy; blood type; tumor location; CA125, TG, LDL, HGB, D-D, and INR; and postoperative electrolyte levels K^+^, Cl^−^, and Ca^2+^ (*P* value < 0.05; Table 1, Supplementary Fig. 1).

**Table 1:** Univariate logistic regression analysis results with ORs and 95% confidence intervals for each predictor.

Variables	Type	Beta	SE	OR (95% CI)	*P*-value
Age		0.126	0.02824	1.134(1.077–1.203)	< 0.001
Blood transfusion volume		0.002	0.00082	1.002(1.001–1.004)	0.004
Operation type					
	Laparoscopic	Reference			
	Open	1.163	0.47001	3.201(1.352–8.824)	0.013
Tumor location					
	Cardia	Reference			
	Body	− 0.541	0.49237	0.582 (0.227–1.612)	0.272
	Antrum	− 1.172	0.5383	0.31(0.107–0.917)	0.029
Blood type					
	A	Reference			
	B	1.249	0.60478	3.486(1.134–12.97)	0.039
	AB	− 14.928	972.3377	3.285 (5.978–95,442)	0.988
	O	1.259	0.57847	3.523(1.232–12.64)	0.029
CA125		0.02	0.00834	1.02(1.001–1.037)	0.019
TG		− 1.374	0.50528	0.253(0.086–0.621)	0.007
LDL		− 0.763	0.27571	0.466(0.267–0.789)	0.006
HGB		− 0.033	0.01001	0.968(0.949–0.987)	0.001
D-D		0	0.00004	1(1–1)	0.026
INR		3.069	1.20121	21.53(1.783–239.9)	0.011
K^+^		− 1.388	0.51167	0.25(0.089–0.661)	0.007
Cl^−^		0.177	0.05425	1.194(1.074–1.33)	0.001
Ca^2+^		− 5.764	1.59846	0.003(0–0.033)	< 0.001

*GC* Gastric Cancer, *SE* Standard Error, *OR* Odds Ratio, *CI* Confidence Interval, *TG* Triglycerides, *LDL* Low-Density Lipoprotein, *HGB* Hemoglobin, *D-D* D-Dimer, *INR* International Normalized Ratio.

**Figure 2:**
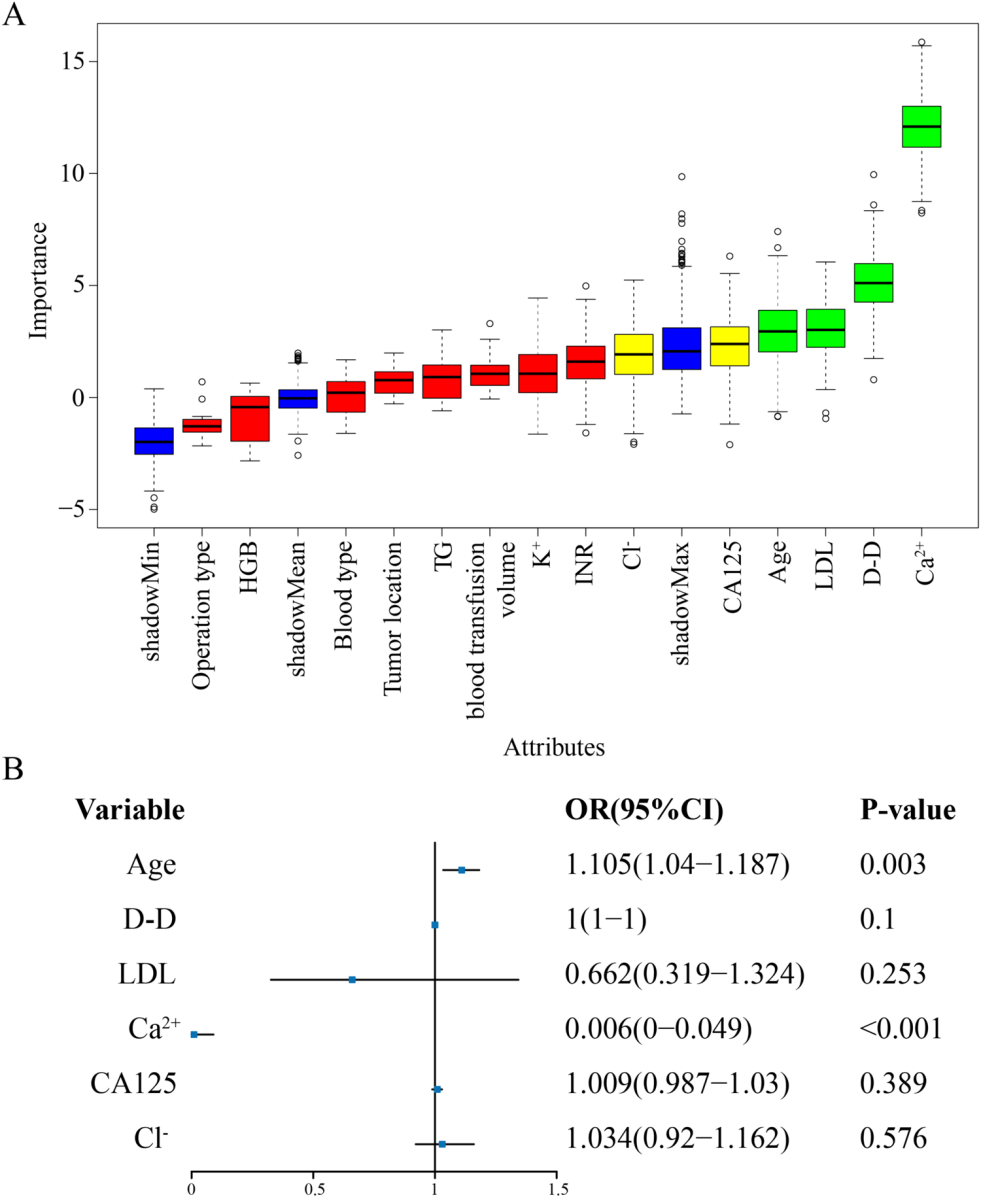
Selection of important clinical factors for lower extremity DVT after radical gastrectomy. (**A**) The results of selected clinical factor analyses using the Boruta algorithm. (**B**) The results of multivariate logistic regression analysis.

**Figure 3:**
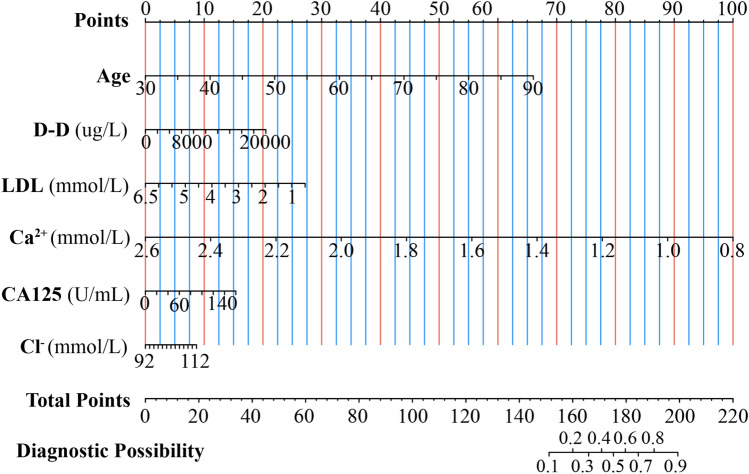
A predictive nomogram was developed using variables from multivariate logistic regression analysis. In the nomogram chart, every variable is allocated a score on the top scoring line, typically ranging from 0 to 100 points. The aggregate score, obtained by adding the points for each variable, aligns with a distinct risk prediction value indicated on the bottom risk line.

To further filter the critical clinical variables, we employed the Boruta algorithm. The main objective of the Boruta algorithm is to identify the truly important features from a given set of features and filter out those that do not have a significant impact. As shown in Fig. 2A, six significant clinical factors were selected after 500 iterations, namely, age, D-D, LDL, CA125, Ca^2+^, and Cl^−^. Using multivariate logistic regression, we analyzed six key variables highlighted by the above analysis. The findings indicated that factors such as age and Ca^2+^ independently contributed to lower extremity DVT after radical gastrectomy in patients with GC, as detailed in Fig. 2B. Additionally, a nomogram was developed using the outcomes from the multivariate logistic regression analysis. The individual risk scores assigned to each element within the nomogram are presented in Fig. 3. A higher score indicates an increased likelihood of lower extremity DVT after radical gastrectomy.

### The predictive performance of our model

**Figure 4:**
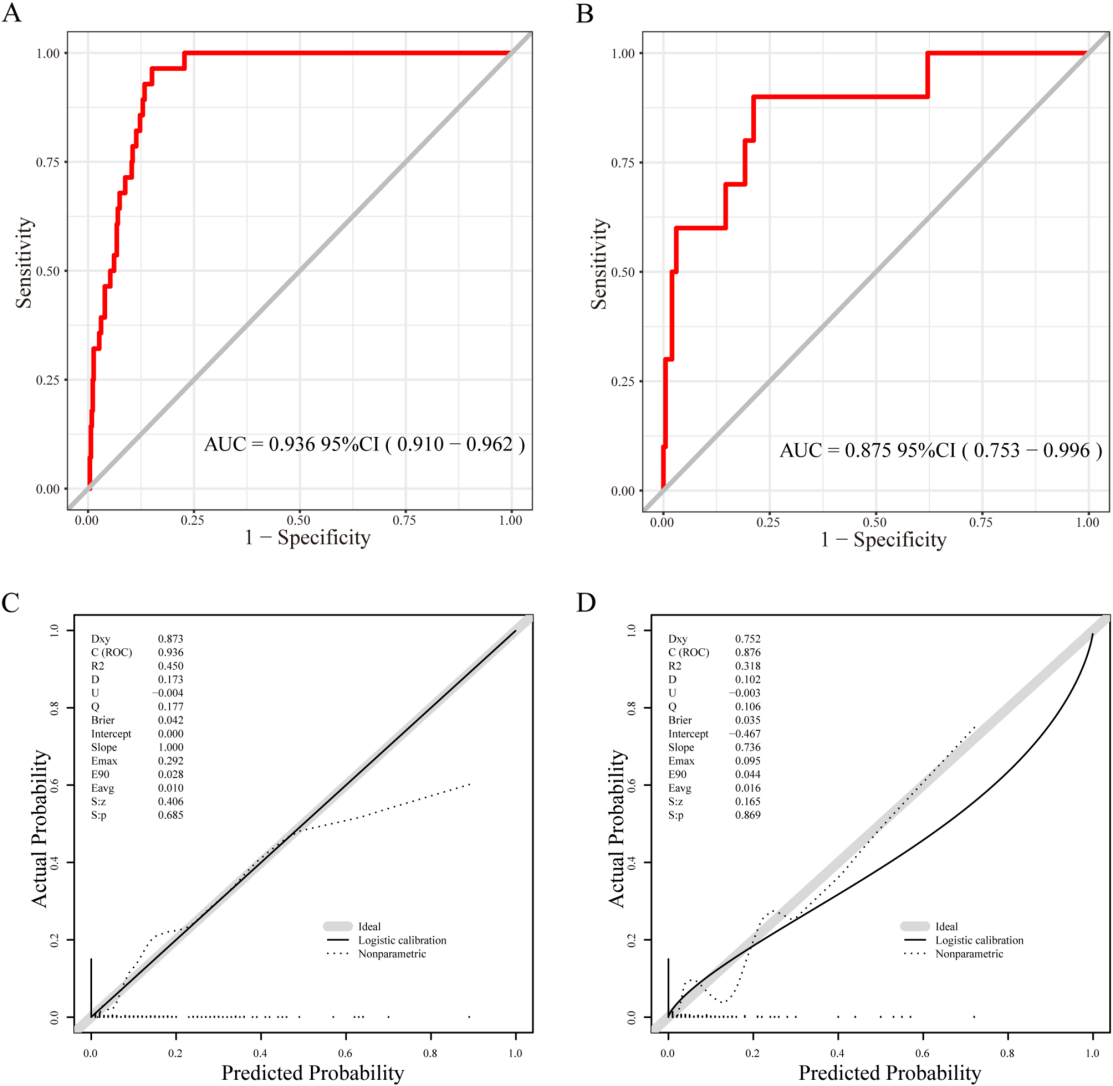
The ROC and calibration plot of our model in the training and validation sets. (**A**) The ROC curve of the training set. (**B**) The ROC curve of the validation set. (**C**) The calibration plot of the training set. (**D**) The calibration plot of the validation set.

**Figure 5:**
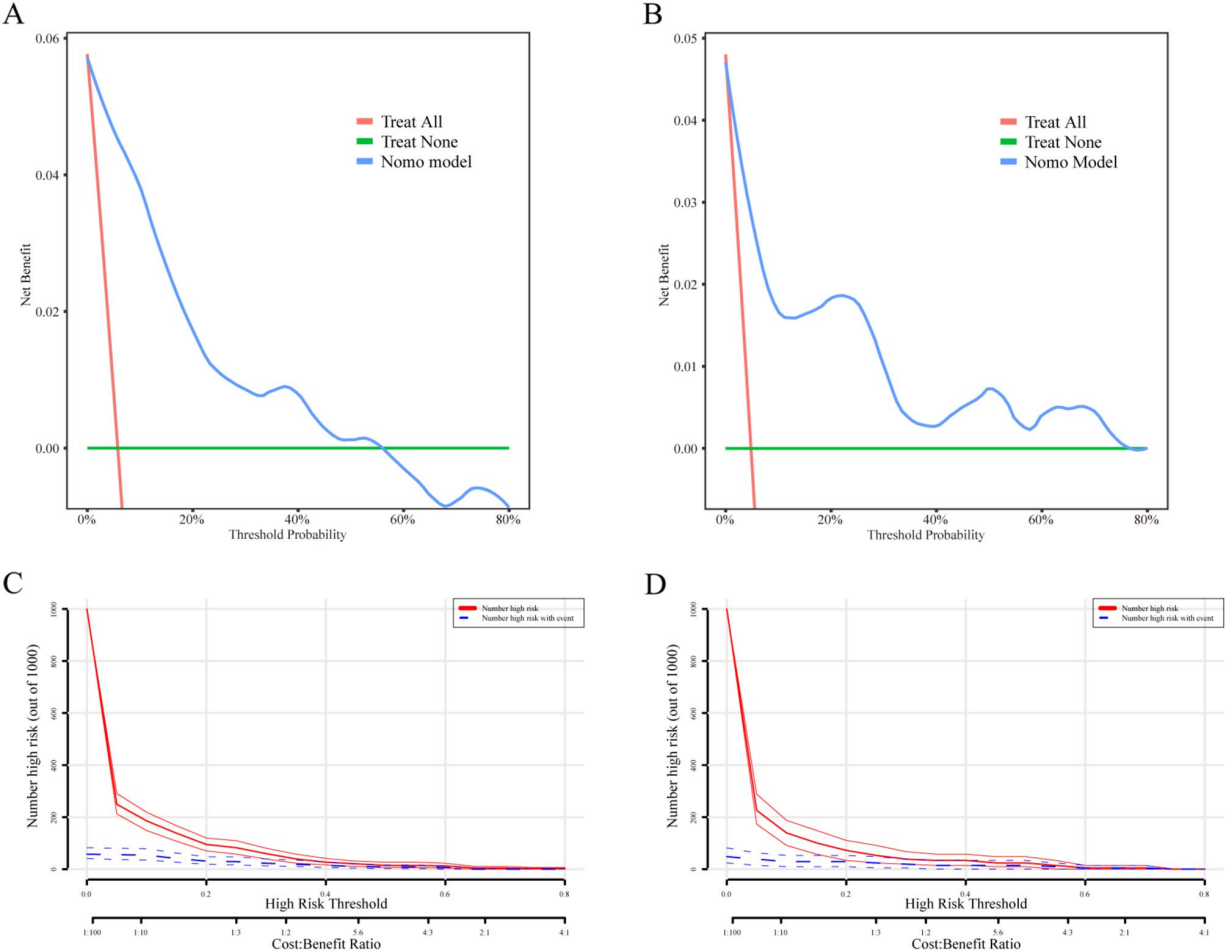
DCA and CIC analysis of the nomogram. (**A**) DCA of the training cohort. (**B**) DCA in the validation set. (**C**) CIC analysis in the training cohort. (**D**) CIC analysis in the validation cohort.

The predictive model demonstrated an AUC of 0.936 (95% CI, 0.910–0.962) in the training cohort (Fig. 4A). Moreover, the AUC of the nomogram was 0.875 in the validation cohort (95% CI, 0.753–0.996; Fig. 4B). The calibration plot and Hosmer–Lemeshow test (*P* value > 0.05) indicated good agreement between the predicted and observed probabilities of DVT development in both the training and validation sets (Fig. 4C,D). In the clinical application of our nomogram, DCA was conducted, and the results suggested that our nomogram offered greater net benefit (Fig. 5A,B). Finally, we conducted a CIC analysis, as illustrated in Fig. 5C,D, to assess the practical utility and overall benefit of our top-performing model. The CIC analysis clearly demonstrated that the nomogram effectively enhanced decision-making across various threshold probabilities, highlighting the significant predictive accuracy of our model. Additionally, the effectiveness of the nomogram was compared with that of the traditional D-D level using metrics such as the NRI and IDI. The results verified the superior performance of the nomogram over the traditional D-D (NRI = 1.526, *P* value < 0.001; IDI = 0.240, *P* value < 0.001). Additionally, the performance of the ROC curve in our model was better than that of D-D, the INR, APTT, PT and Fib (AUC = 0.711 in the training set and 0.727 in the validation set; Supplementary Fig. 2).

### Model evaluation of multiple machine learning methods

**Figure 6:**
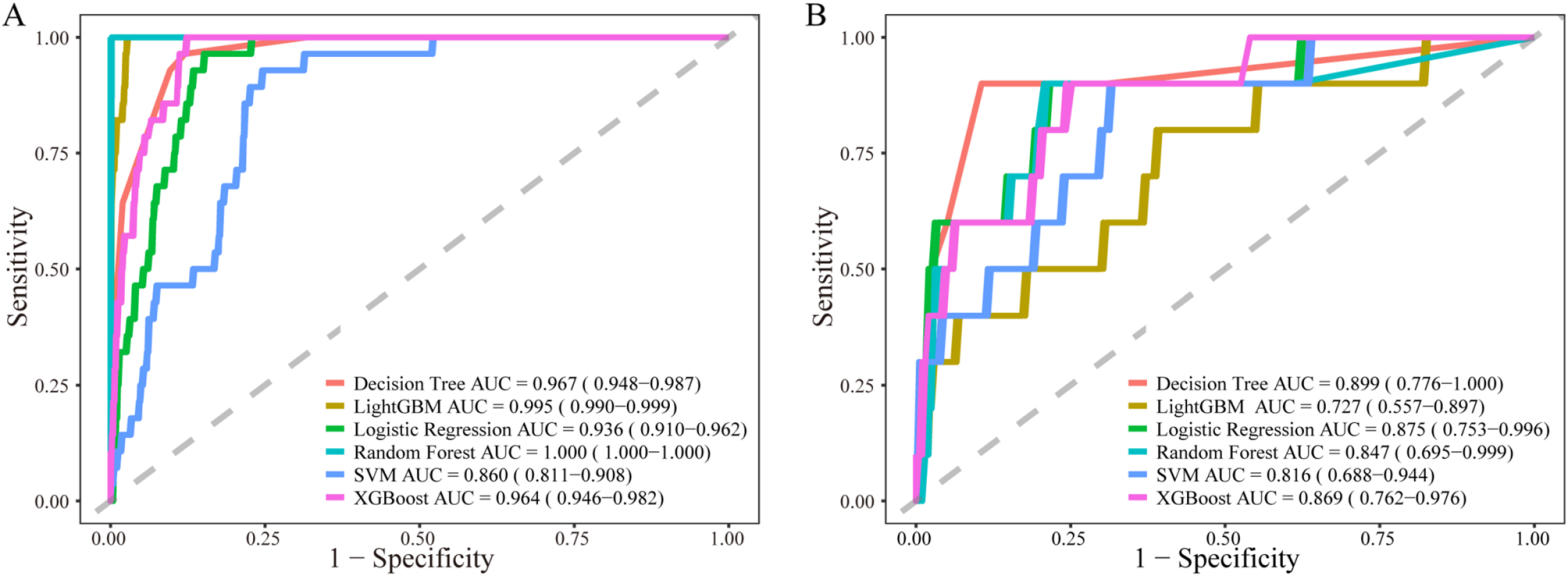
The ROC curves of multiple machine learning methods. (**A**) ROC curve in the training set. (**B**) ROC curve in the validation set.

To demonstrate the significance of the variables we screened and the predictive value of our model, we subsequently employed various machine learning algorithms for evaluation. First, we generated a decision tree algorithm, which suggested good predictive power in both the training and validation sets. Similar outcomes were also observed for the random forest, SVM, XGBoost, and LightGBM models in training and validation sets (Fig. 6A,B). The results of the various machine learning algorithms indicate that our model possesses strong predictive value.

## Discussion

The significance of this study in addressing the risk of lower extremity DVT in postoperative GC patients is underscored by the substantial morbidity and potential mortality associated with VTE in this patient population^13^. Notably, GC surgery is linked to a heightened risk of postoperative VTE, including DVT and PE^14,15^. Compared with air wave pressure therapy instrument, rivaroxaban has better preventive effect on lower extremity DVT after GC operations^16^. A systematic review and meta-analysis involving 111,936 patients indicated that the 1-month incidence of VTE post GC surgery was 1.8%, and specifically for DVT, it was 1.2%^11^. Among the 666 Korean patients after gastrectomy, the overall incidence of VTE was 2.1%^17^. These figures highlight the critical importance of focusing on DVT in GC patients postsurgery. Moreover, this study aims to fill a significant gap in the current research. While the incidence of VTE in GC patients is known, there is less focus on predicting lower extremity DVT, specifically in the postoperative phase of GC. A retrospective cohort study revealed that age, preoperative blood glucose level, postoperative anemia, and tumor malignancy were independent risk factors for postgastrectomy VTE in GC patients^18^. However, compared with previous studies, our study focused on predictive modeling using a comprehensive set of clinical indicators, including age and calcium ion levels, and provided a more detailed risk assessment tool; this underscores the need for predictive models that can accurately identify patients at higher risk for DVT following GC surgery, enabling targeted prophylactic strategies.

The predictive model developed in this study demonstrated high accuracy, as reflected by the area under the curve (AUC) values in both the training and validation sets. This finding indicates the strong predictive capability of the NRS-2002, which is essential in clinical settings for risk stratification and management of DVT in postoperative GC patients. The importance of such predictive models is highlighted by the varying risk factors identified across different studies, including age and tumor-related factors. Age has been consistently identified as a significant risk factor for postoperative VTE^18^, and the role of calcium in coagulation processes further substantiates its relevance as a predictive marker in the developed model. These factors provide critical insights into patient-specific risk profiles and can guide clinicians in the prophylaxis and management of DVT after GC surgery.

According to our univariate analysis, age emerged as a significant independent variable influencing DVT occurrence following gastrectomy in GC patients. Furthermore, multivariate analysis highlighted age as a contributing factor to the development of postoperative DVT in these patients. Age is also a risk factor for VTE in patients with GC^19^. Here, we found that calcium ions were a significant clinical factor in our model. The role of calcium ions in the coagulation process and thrombosis is complex and multifaceted; one key aspect is their involvement in platelet activation. Platelets play a critical role in maintaining hemostasis and vessel integrity under normal conditions and in thrombosis under pathological conditions. The activation of platelets strongly depends on an increase in the intracellular calcium (Ca^2+^) concentration. This increase results from the release of Ca^2+^ by the dense tubular system and the entry of Ca^2+^ from the extracellular space^20^. In the context of fibrinogen clotting, calcium ions are also known to be necessary for the normal polymerization of fibrin monomers^21^. In the activation of coagulation factor XIII, an important player in the final stages of the coagulation cascade, calcium also plays a crucial role^22^. Therefore, calcium ions are integral to the coagulation process and influence various stages, from platelet activation to stabilization of the fibrin clot.

LDL plays a significant role in the pathogenesis of atherothrombotic processes. It can modify the antithrombotic properties of the vascular endothelium and influence vessel contractility, partly by reducing the availability of endothelial nitric oxide and activating proinflammatory signaling pathways. These modified intravascular LDLs promote the formation of foam cells from smooth muscle cells and macrophages, increasing the vulnerability of atherosclerotic plaques and enhancing the thrombogenicity of both plaques and blood^23^.

Several research findings indicate that a reduction in hemoglobin levels may serve as an indicator of increased VTE risk and poorer prognosis in cancer patients^5^. Another study demonstrated that low hemoglobin levels at baseline correlated with an increased likelihood of symptomatic VTE, symptomatic DVT, and nonfatal PE^24^. Another study investigated the influence of anemia on the risk of bleeding in patients receiving anticoagulant therapy for VTE^25^. These findings underscore the importance of considering anemia as a factor in the management of VTE, particularly in populations at high risk, such as acutely ill patients and those with cancer.

Different from previous research studies, here, we collected plentiful and comprehensive clinical indicators including a total of 47 baseline, preoperative, surgical and pathological clinical data. So far, we have included the largest number of clinical variables in our study. Most importantly, in our research, we use a variety of comprehensive machine learning algorithms. Machine learning methods have been successfully applied in various fields of medicine and have shown great potential in predictive data analytics^26^. Compared to conventional prediction models (logistic regression), machine learning models perform as well as logistic regression models; however, some machine learning methods exhibit exceptional performance^27^. One study developed machine learning models (LightGBMs) to predict VTE diagnosis and 1-year risk using electronic health record data from diverse populations. These tools outperformed existing risk assessment tools, showing robust performance across various VTE types and patient demographics^28^. In our study, we used various machine learning algorithms, including logistic regression, decision trees, random forests, SVM, XGBoost, and LightGBM. By applying these insights to our study, we can anticipate a more robust and precise model for predicting lower extremity DVT risk in postoperative GC patients, potentially leading to better patient outcomes.

In a real-world setting, the model could be integrated into clinical decision-making processes, perhaps through electronic health records systems. By inputting patient-specific data, health care providers could receive immediate risk assessments, guiding them in choosing the most appropriate prophylactic measures. This approach aligns with the growing trend of personalized medicine, where treatment and preventive strategies are tailored to individual patient characteristics and risk profiles.

Despite its contributions, one potential limitation of this study is its retrospective nature, which may introduce biases such as selection bias or information bias. The data used in the study might also have limitations in terms of their scope or the accuracy of the recorded information. Another limitation could be the generalizability of the findings. The study’s results are based on a specific patient population and may not be directly applicable to other populations or settings. Additionally, this study developed a population-specific predictive model. However, the selected predictors were not unique to any specific population, as they appear applicable to patients undergoing gastrointestinal, liver, and pancreatic surgeries. Therefore, it raises the question of whether it is necessary to develop a postoperative lower limb thrombosis prediction model specifically for patients undergoing radical gastrectomy.

Future research should focus on validating the predictive model in diverse patient populations and clinical settings to enhance its generalizability. Future studies could also explore the integration of the model into clinical workflows and its impact on patient outcomes in a real-world setting. However, further research is needed to understand the biological mechanisms underlying the identified risk factors for DVT in GC patients; this could lead to more targeted therapeutic interventions. Additionally, incorporating new types of data, such as genetic or molecular marker data, could improve the model’s predictive accuracy.

In summary, the development of a predictive model for lower extremity DVT in postoperative GC patients addresses a vital clinical need. The model’s accuracy and ability to identify significant predictive factors make it a valuable tool for enhancing postoperative care and patient outcomes in patients with GC.

### Supplementary Information

Supplementary Figures.

Supplementary Tables.

## Data Availability

All data generated or analyzed during this study are included in this published article and supplementary files.

## References

[1] Rawla P, Barsouk A (2019). Epidemiology of gastric cancer: Global trends, risk factors and prevention. Prz Gastroenterol..

[2] Smyth EC, Nilsson M, Grabsch HI, van Grieken NC, Lordick F (2020). Gastric cancer. Lancet.

[3] Lyman GH, Carrier M, Ay C, Di Nisio M, Hicks LK, Khorana AA (2021). American Society of Hematology 2021 guidelines for management of venous thromboembolism: Prevention and treatment in patients with cancer. Blood Adv..

[4] Donnellan E, Khorana AA (2017). Cancer and venous thromboembolic disease: A review. Oncologist.

[5] Majmudar K, Golemi I, Tafur AJ, Toro JD, Visona A, Falga C (2020). Outcomes after venous thromboembolism in patients with gastric cancer: Analysis of the RIETE Registry. Vasc. Med..

[6] Khorana AA, Mackman N, Falanga A, Pabinger I, Noble S, Ageno W (2022). Cancer-associated venous thromboembolism. Nat. Rev. Dis. Primers.

[7] Rodrigues CA, Ferrarotto R, Kalil Filho R, Novis YA, Hoff PM (2010). Venous thromboembolism and cancer: A systematic review. J. Thromb. Thrombolysis.

[8] Stricker H (2014). Venous thromboembolism and cancer: pathophysiology and incidence. Vasa.

[9] Osaki T, Saito H, Fukumoto Y, Kono Y, Murakami Y, Shishido Y (2018). Risk and incidence of perioperative deep vein thrombosis in patients undergoing gastric cancer surgery. Surg. Today.

[10] Xu Q, Lei H, Li X, Li F, Shi H, Wang G (2023). Machine learning predicts cancer-associated venous thromboembolism using clinically available variables in gastric cancer patients. Heliyon..

[11] Xiang L, Jin S, Yu Y, Wang D, Chen H (2023). Risk of venous thromboembolism in patients undergoing gastric cancer surgery: A systematic review and meta-analysis. BMC Cancer..

[12] Falanga A, Ay C, Di Nisio M, Gerotziafas G, Jara-Palomares L, Langer F (2023). Venous thromboembolism in cancer patients: ESMO clinical practice guideline. Ann. Oncol..

[13] Fuentes HE, Oramas DM, Paz LH, Wang Y, Andrade XA, Tafur AJ (2018). Venous thromboembolism is an independent predictor of mortality among patients with gastric cancer. J. Gastrointest. Cancer..

[14] Khan F, Tritschler T, Kahn SR, Rodger MA (2021). Venous thromboembolism. Lancet.

[15] Cohen AT, Katholing A, Rietbrock S, Bamber L, Martinez C (2017). Epidemiology of first and recurrent venous thromboembolism in patients with active cancer. A population-based cohort study. Thromb. Haemost..

[16] Dong Q, Zhu X, Gao Y, Wang Z, Zheng D, Zhu J (2022). Analysis of the preventive action of rivaroxaban against lower extremity deep venous thrombosis in patients after laparoscopic radical gastrectomy. Comput. Math. Methods Med..

[17] Jung YJ, Seo HS, Park CH, Jeon HM, Kim JI, Yim HW (2018). Venous thromboembolism incidence and prophylaxis use after gastrectomy among korean patients with gastric adenocarcinoma: The PROTECTOR randomized clinical trial. JAMA Surg..

[18] Li XP, Wang YY, Sun YS, Zhang LJ, Zhao XY, Liu ZQ (2023). Preoperative and postoperative clinical signatures of postgastrectomy venous thromboembolism in patients with gastric cancer: A retrospective cohort study. Asian J. Surg..

[19] Tanizawa Y, Bando E, Kawamura T, Tokunaga M, Makuuchi R, Iida K (2017). Prevalence of deep venous thrombosis detected by ultrasonography before surgery in patients with gastric cancer: A retrospective study of 1140 consecutive patients. Gastric Cancer.

[20] Davlouros P, Xanthopoulou I, Mparampoutis N, Giannopoulos G, Deftereos S, Alexopoulos D (2016). Role of calcium in platelet activation: Novel insights and pharmacological implications. Med. Chem..

[21] Furlan M, Steinmann C, Jungo M, Lammle B (1996). Binding of calcium ions and their effect on clotting of fibrinogen Milano III, a variant with truncated A alpha-chains. Blood Coagul. Fibrinolysis..

[22] Turner BT, Maurer MC (2002). Evaluating the roles of thrombin and calcium in the activation of coagulation factor XIII using H/D exchange and MALDI-TOF MS. Biochemistry.

[23] Badimon L, Vilahur G, Padro T (2009). Lipoproteins, platelets and atherothrombosis. Rev. Esp. Cardiol..

[24] Chi G, Gibson CM, Hernandez AF, Hull RD, Kazmi SHA, Younes A (2018). Association of anemia with venous thromboembolism in acutely Ill hospitalized patients: An APEX trial substudy. Am. J. Med..

[25] Kuperman A, Lopez-Reyes R, Bosco LJ, Lorenzo A, Jose B, Farge Bancel D (2018). Anemia and bleeding in patients receiving anticoagulant therapy for venous thromboembolism. J. Thromb. Thrombolysis..

[26] Jin MC, Rodrigues AJ, Jensen M, Veeravagu A (2022). A discussion of machine learning approaches for clinical prediction modeling. Acta Neurochir. Suppl..

[27] Song X, Liu X, Liu F, Wang C (2021). Comparison of machine learning and logistic regression models in predicting acute kidney injury: A systematic review and meta-analysis. Int. J. Med. Inform..

[28] Chen R, Petrazzini BO, Malick WA, Rosenson RS, Do R (2024). Prediction of venous thromboembolism in diverse populations using machine learning and structured electronic health records. Arterioscler. Thromb. Vasc. Biol..

