# Supplementary material for: Predictive modeling of lower extreme deep vein thrombosis following radical gastrectomy for gastric cancer: based on multiple machine learning methods
Source: Sci Rep. 2024 Jul 8;14:15711. doi: 10.1038/s41598-024-66754-y (PMC11231254; doi:10.1038/s41598-024-66754-y)
Supplement: Supplementary file 1 — Supplementary Figures. [file 41598_2024_66754_MOESM1_ESM.docx]

**Supplementary Figures**


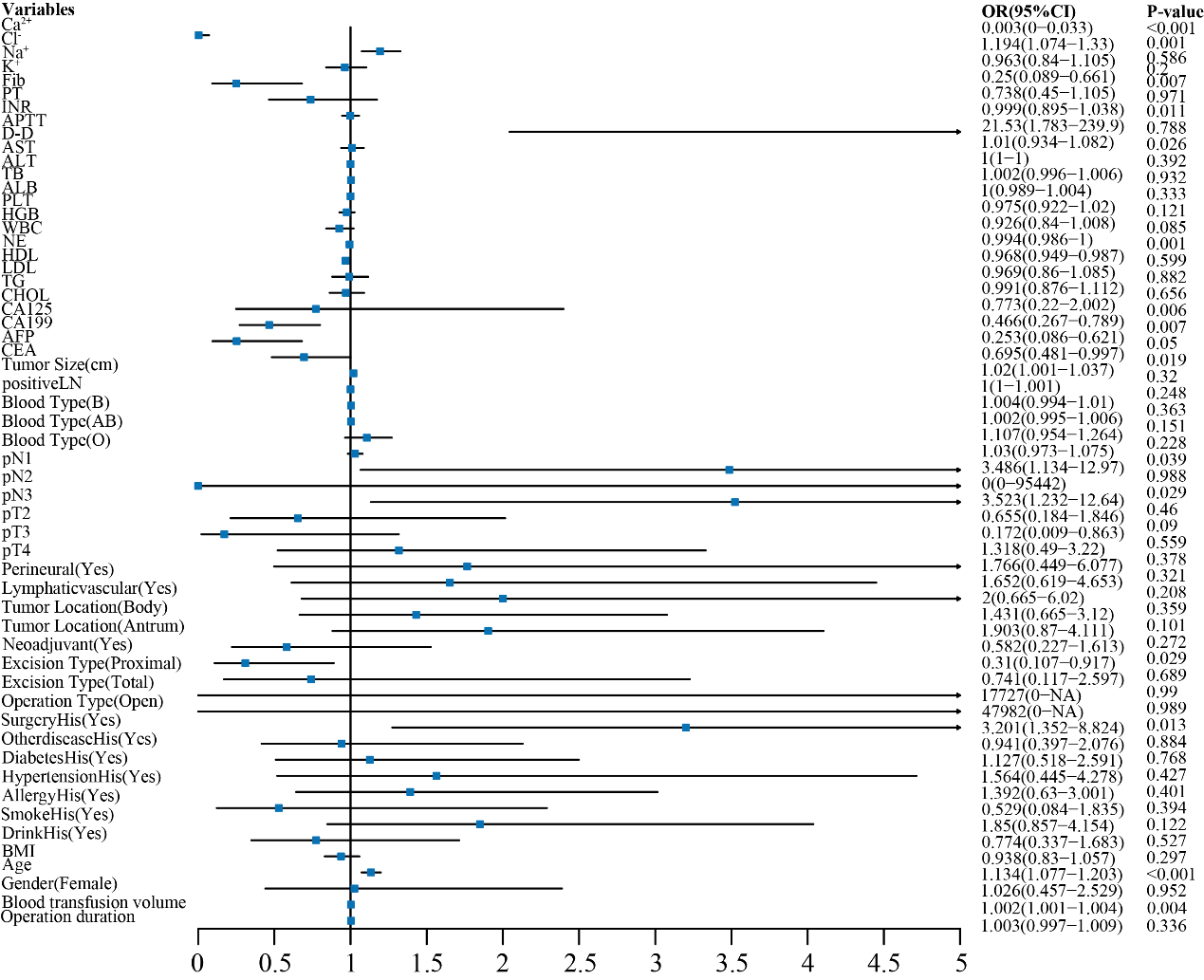


**Supplementary Figure 1. The results of univariate logistic analysis were visualized using a forest plot.**


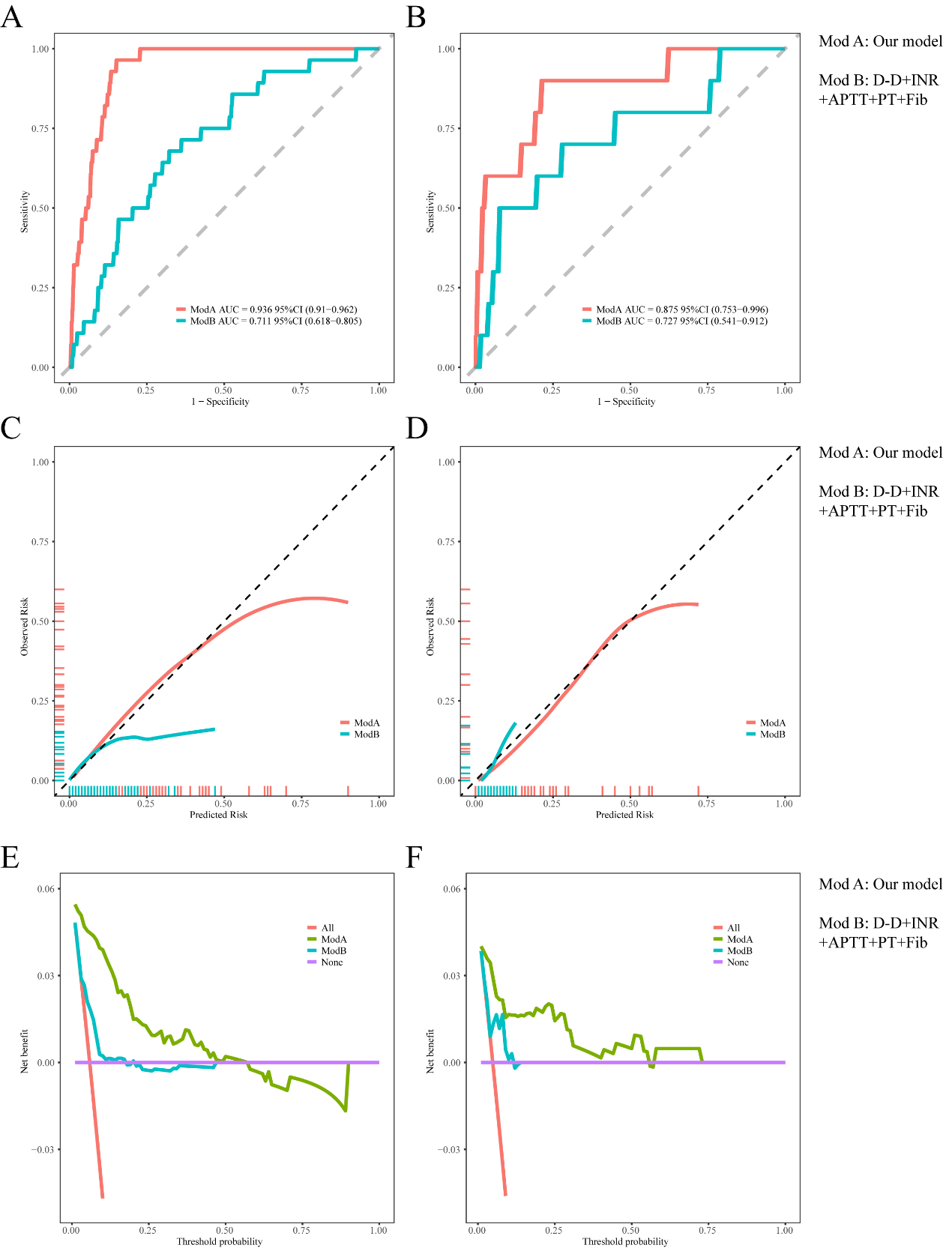


**Supplementary Figure 2. The ROC curve, calibration plot and DCA for multiple model comparisons.** (A) ROC curve of multiple model comparisons in the training set. (B) ROC curve of multiple model comparisons in the validation set. (C) The calibration plot of multiple model comparisons in the training set. (D) The calibration plot of multiple model comparisons in the validation set. (E) DCA of multiple model comparisons in the training set. (F) DCA of multiple model comparisons in the validation set.

**Supplementary Table Legends**

**Supplementary Table 1. Comparison of clinical baseline data between the training and validation sets.**
